# Highly organised and dense vertical silicon nanowire arrays grown in porous alumina template on <100> silicon wafers

**DOI:** 10.1186/1556-276X-8-287

**Published:** 2013-06-17

**Authors:** Therese Gorisse, Ludovic Dupré, Pascal Gentile, Mickael Martin, Marc Zelsmann, Denis Buttard

**Affiliations:** 1 CNRS/UJF-Grenoble1/CEA LTM, 17 rue des Martyrs, Grenoble 38054, France; 2 SiNaPS Lab - SP2M, UMR-E CEA/UJF-Grenoble 1, INAC, Grenoble 38054, France; 3 Université Joseph Fourier/IUT-1, 17 quai C. Bernard, Grenoble 38000, France

**Keywords:** Anodic alumina oxide, Nanoimprint lithography, Templates, Chemical vapour deposition, Nanowires, Silicon, Hexagonal array, Defect-free

## Abstract

In this work, nanoimprint lithography combined with standard anodization etching is used to make perfectly organised triangular arrays of vertical cylindrical alumina nanopores onto standard <100>−oriented silicon wafers. Both the pore diameter and the period of alumina porous array are well controlled and can be tuned: the periods vary from 80 to 460 nm, and the diameters vary from 15 nm to any required diameter. These porous thin layers are then successfully used as templates for the guided epitaxial growth of organised mono-crystalline silicon nanowire arrays in a chemical vapour deposition chamber. We report the densities of silicon nanowires up to 9 × 10^9^ cm^−2^ organised in highly regular arrays with excellent diameter distribution. All process steps are demonstrated on surfaces up to 2 × 2 cm^2^. Specific emphasis was made to select techniques compatible with microelectronic fabrication standards, adaptable to large surface samples and with a reasonable cost. Achievements made in the quality of the porous alumina array, therefore on the silicon nanowire array, widen the number of potential applications for this technology, such as optical detectors or biological sensors.

## Background

Low-cost and versatile fabrication of functional nanostructures, for example for nanowires, nanocrystals or nanotubes, becomes of great importance in an increasing number of potential commercial devices [1-6]. In this context, the general approach of directed self-assembly (DSA) seems to be favoured by a high number of scientists and engineers because it uses natural properties and top-down methods to create nanostructures already positioned and organised. As an example, DSA was introduced in the International Technology Roadmap for Semiconductors in 2007. The most common DSA approach consists of organising di-block copolymer features [7] in lithographically created topographical [8] or chemical [9] templates. Another promising DSA approach is the use of anodic aluminium oxide (AAO) as templates for the growth of nanoobjects [10]. An electrochemical oxidation of aluminium in acid solutions will naturally produce a highly dense, roughly triangular array of nanopores in alumina [11]. By varying experimental parameters as acid electrolyte, the applied voltage or the anodization time, geometrical characteristics of the porous membrane can be adjusted. In particular, the diameter, the depth of pores or the distance between nearest neighbours can be tuned. Periodicity of the fabricated arrays is in the range of 80 to 460 nm, and pore diameters are in the range of 15 to 100 nm [12]. Compared to the di-block copolymer DSA approach, AAO presents the advantage of very high aspect ratio features with no real limitation. Besides, due to its high thermal and mechanical resistance, the AAO matrix allows additional processing steps, therefore enabling its integration in functional devices.

Consequently, this material is a good candidate for the fabrication of organic, inorganic or metallic nanostructures [13,14]. These nanostructures offer a very large panel of applications including among others data storage with ferroelectric materials [1], sensors [2] and supercapacitors [3]. More specifically, porous AAO can be used to guide the growth of mono-crystalline nanowires by chemical vapour deposition (CVD). This system is useful for photovoltaic purpose [4], optical detectors [5] or biochemical captors [6]. However, until now, very few references report the use of AAO for the growth of these nanoobjects, and it is the conventional methods to produce AAO, so-called simple or double anodization [10,15], which have been employed [4,16]. With this technique, the hexagonal order is maintained only on domains of few square micrometres, a sacrificial layer of aluminium is lost and the pore’s size and shape distribution is high [17]. These limitations lead obviously to a reduction in the performance of later devices or a decrease in the number of potential applications [18].

To improve the control of formation of AAO arrays, various top-down methods have been proposed in the literature to pre-pattern the aluminium surface prior to the electrochemical treatment such as focused ion beam lithography [19,20], holographic lithography [21], block copolymer micelles [22], soft imprinting [23], mould-assisted chemical etching [24], colloidal lithography [25], nanoindentation [26,27], nanoimprint lithography (NIL) [1,28] and guided electric field [29]. Such directed assembly approaches are not only very interesting in terms of pores positioning and control of pore’s size distribution, but also allow the use of a thin initial aluminium layer -micrometre scale- supported by a silicon wafer [30]. Among all top-down guiding methods, NIL is very promising. Indeed, it is the only approach that allows working with perfectly organised arrays at wafer scale and at reasonable cost. Though it is generally prepared with expensive exposure tools like electron-beam lithography, the mould can be reused a very large number of times [31]. Also, compared to nanoindentation, the use of an intermediate resist transfer layer permits to work with fragile substrates, for example with already processed wafers. At last, NIL is perfectly adapted to the already existing microelectronic processing tools.

In this work, we present our results on the use of NIL-guided AAO templates for the fabrication of highly regular and dense epitaxial silicon nanowire arrays grown perpendicularly to <100> silicon substrates and on surfaces as large as 4 cm^2^. The originality resides in the quality of the array obtained and in the choice of low-cost and large-scale technologies to achieve this quality. We present the used routes and discuss the improvements made compared to other existing methods.

## Methods

**Figure 1:**
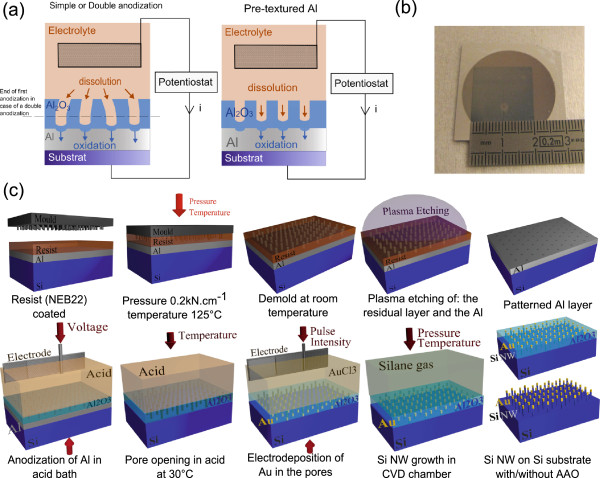
**General schematic of the process steps used. (a)** Porous alumina layer fabrication using electrochemistry. **(b)** Picture of a 2 × 2-cm^2^ sample, reference: centimetre scale. **(c)** Thermal nanoimprint lithography process used to pattern the surface of thin aluminium layers supported by silicon substrates, Al anodization and Si NW growth.

Porous aluminium oxide is naturally obtained by anodizing aluminium in an acid bath. During anodization, two competing phenomena occur simultaneously: oxidation of the aluminium layer and dissolution of the alumina. Although this phenomenon is still not fully understood [32], the dissolution is first localised mainly on surface defects, for example grain boundaries, and then at the bottom of the pores. Both oxidation and dissolution lead to the growth of a porous Al_2_O_3_ layer as described in Figure 1a. During anodization, a constant thickness of Al_2_O_3_, called a barrier layer [33], is kept under the pores. The thickness of the barrier layer is proportional to the applied voltage. Due to this specific property, pores will naturally grow with an inter-pore distance equal to two times the barrier layer [34]. Thus, the pores will slowly organise in-plane in a hexagonal array during the alumina growth. Period *a* of the array depends linearly with the applied voltage *V*; Equation 1 shows the value obtain with the set-up developed in our laboratory.

(1) a=2.31*V

**Figure 2:**
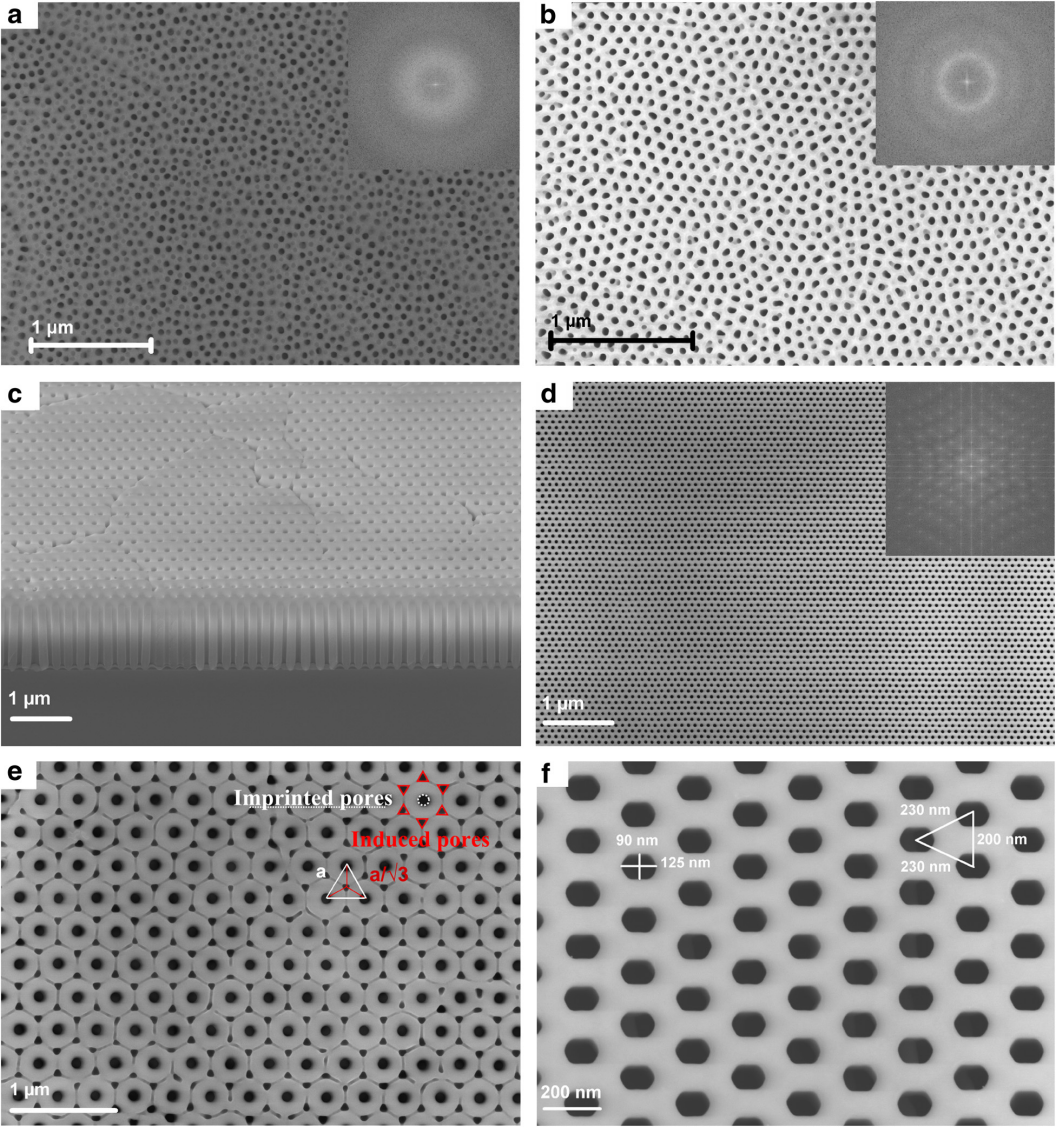
**Scanning electron micrographs of porous alumina.** (**a**) Simple anodization in oxalic acid at 40 V; insert: fast Fourier transform of the scanning electron microscopy (SEM) image. (**b**) Double anodization in oxalic acid at 40 V; insert: fast Fourier transform of the SEM image. (**c**) Cross-sectional view before widening and opening of the pore’s end with a lattice constant of 250 nm. (**d**) Top view after widening and opening of the pore’s end with a lattice constant of 100 nm; insert: fast Fourier transform of the SEM image. (**e**) Example of pore array obtained when only one third of the pores are localised using nanoimprint lithography. (**f**) Example of non-cylindrical pore array obtained with non-equilateral triangular lattice.

Direct oxidation of Al, also called simple anodization described in Figure 1a [15], leads to a poor organisation in particular at the surface, shown in Figure 2a. To improve the organisation, a process, called double anodization, was proposed [10]. A sacrificial layer of aluminium is oxidised in which the pores arrange in a hexagonal array as presented in Figure 1a. Afterwards, this oxide layer is removed. An organised array of pits is left at the aluminium surface because of the rounded shape of the bottom of the pores. It is used to guide the pores in the second anodization process. Nevertheless, using this approach, a long-range order is maintained only on domains of few square micrometres, as depicted in Figure 2a,b, and part of the aluminium layer is lost due to the first sacrificial anodization.

In the case of thermal NIL presented here, holes are pre-patterned in a triangular array on the surface of a thin aluminium layer deposited on a P+ conductive Si wafer. As described in Figure 1c, a thermoplastic resin (NEB22 from Sumitomo Chemicals, Tokyo, Japan) is coated on an aluminium layer. A silicon mould, treated with an anti-sticking layer [31] and presenting a triangular array of pits, is then pressed on the sample in an EVG520 hot embossing tool (EV Group, St. Florian an Inn, Austria) at 0.2 kN.cm^−2^ and 125°C. Mould patterns are reproduced in the polymer since the applied temperature is higher than the resin's glass transition temperature. After removal from the mould at room temperature, the pattern is transferred into the surface of the Al layer using a conventional plasma dry etching technique. In a Centura 5200 reactive ion etching chamber (Applied Materials, Santa Clara, CA, USA), a Cl_2_/Ar/O_2_ plasma is used to remove the residual resin layer and a Cl_2_/BCl_3_ plasma is used for etching the Al surface. The final structure consists of a 2 × 2-cm^2^ surface of aluminium structured with holes of few nanometres depth in a triangular array of different periods according to the initial mould design. During the anodization, these holes will act as surface defects, initiating the pore growth as described in Figure 1a. The layer is so directly anodized at the voltage corresponding to the period given by the NIL and according to Equation 1. Samples are anodized in a home-made cell under a constant voltage, in an orthophosphoric or oxalic acid bath at constant temperature (*T* = 8°C). The electrolyte is stirred during the process of anodization to facilitate the flow of the species in the electrolyte and to remove the bubbles of H_2_ gas from the platinum electrode. A Parstat 2273 potentiostat (Princeton Applied Research, Oak Ridge, TN, USA) is used to apply a constant voltage and to follow the *I*-*V* curve *in situ* between a platinum circular electrode and the sample. In order to obtain defect-free triangular arrays of pores, the voltage has to be adjusted so that the natural period of the porous alumina corresponds to the NIL-fabricated guiding pattern. The anodizing time does not differ significantly from the classical anodization time of a simple anodization: with 3% oxalic acid under 40 V at 8°C, simple anodization of 1 μm of Al takes 1,750 s and anodization after nanoimprint lasts 1,700 s. Furthermore, under these experimental conditions, the ratio between the thickness of Al layer deposited and the final thickness of highly organised alumina is evaluated at 1.25. Figure 1b shows an array of 2 × 2 cm^2^ of highly organised porous alumina.

After the removal of the remaining alumina layer at the bottom of pores with a wet-etching step in orthophosphoric acid as described in Figure 1c, the template is annealed for 15 min at 580°C under argon gas flow in order to improve its mechanical properties. Samples are then cleaned with acetone and isopropanol, and the native silicon oxide layer at the bottom of the pores is removed with hydrofluoric acid (HF) vapour etching. The catalyst, gold or copper, is deposited only at the bottom of the pores on the conductive Si wafer by pulse electrodeposition using a gold chloride or copper sulphate solution. Ions of gold or copper are oxidised on the surface of the silicon wafer until the creation of a thin layer of catalyst. Alumina, being an insulator, prevents all deposition elsewhere, but on the silicon which is present here only at the bottom of the pores. Pulse deposition gives better results than classical electrodeposition because the ions migrate more easily inside the pores till the silicon surface [4]. Nanowires are then grown, using the so-called vapour-liquid–solid (VLS) process [35], in a hot wall low-pressure CVD reactor under a silane flow of 50 sccm and a hydrogen flow (carrier gas) of 1,400 sccm. Temperature is set to 580°C, and pressure was set to 3 Torr. To prevent diffusion of the catalyst, hydrogen chloride is added in the gas flow [36]. The addition of a doping gas, diborane or phosphine, can also be used to obtain P-or N-type doped silicon nanowires [37]. The alumina matrix might be removed after the growth of wires by wet etching in 1% HF, leading to a free silicon array of nanowires as presented in Figure 1c.

## Results and discussion

### Nanoporous alumina templates

Scanning electron microscopy (SEM) images of some of our results are shown in Figure 2c,d. One can notice the regularity of the array of cylindrical pores from the top to the bottom of the alumina layer, the smooth walls of the pores, the homogeneity of the pore shape and diameter. Although the grain boundaries, due to the aluminium deposition, are still visible in Figure 2c, orientation of the organisation is not disturbed over the grains. These Al grain boundaries were removed by improving the Al deposition method; temperature and speed of deposition were optimised. Indeed, Figure 2d shows that there are no more grain boundaries. On fabricated samples, inter-pore distances vary from 90 to 250 nm (Figure 2c shows a period of 250 nm and Figure 2d, 100 nm), and pore sizes vary from 30 to 150 nm. The NIL period is restricted by the fabrication techniques of the mould: the resolution of the e-beam set-up used is limiting the period to 90 nm. The upper limit is related to the anodization voltage: above 200 V, which corresponds to a period of 460 nm, the aluminium is damaged. Typical layer thickness is around 1,250 nm. Array period *a* is controlled by the applied voltage, whereas the control of the pore diameter is ensured by an additional wet-etching step in orthophosphoric acid. This last step also allows the removal of the residual alumina at the bottom of the pores.

The Fourier transforms (FT) of the simple, double and imprinted anodizations are shown in Figure 2a,b,d. For simple anodization, we observe a large ring, whereas the FT of double-anodized alumina shows a less thick and more prominent circle. If a thick ring is typical of a non-spatial organisation and varying inter-pore distances, we verify with the thin ring that a uniform inter-pore distance without any preferred orientation in the organisation is obtained for double-anodized alumina. This confirms the presence of grains with a hexagonal array randomly orientated. On the FT of the SEM image from the nanoimprinted sample, a hexagonal array of fine dots is seen. This confirms the regularity of the arrays in two directions irrespective of grain size. These samples and the analysis of the SEM images show good versatility and improved control of the array in the case of nanoimprint anodization, making AAO a promising template.

In addition, original structures with a mixed growth of NIL-guided pores and generation of naturally guided pores have been developed. The nanoimprint process is used to pre-texture the aluminium surface with pores in a triangular array of period *a*. When the anodization voltage is adapted to an array of period $a/\sqrt{3}$, pores will be created in the holes made with the nanoimprint process, and it will force the creation of new pores in the middle of three imprinted ones. Samples with excellent regularity were obtained on surfaces of 4 cm^2^, as seen in Figure 2e. The shape of these newly created pores, called ‘induced pores’, can be tuned from a triangular to a cylindrical section by changing the acid used and the anodization conditions, whereas ‘imprinted’ ones always present a rounded shape. This technique not only allows to propose original structures but also to get rid of the limitation due to the complexity to produce templates of small period with the standard high-resolution lithography technique, here, electron-beam lithography. This also proves the ability of this technique to eventually restore any missing pore in the initial pattern.

A mould of isosceles triangular lattice (230 × 230 × 200 nm^3^) was also used instead of the classical equilateral triangle. During oxidation, the isosceles lattice is preserved as depicted in Figure 2f. However, we observe pores enlarging in the direction of the apex, leading to an oval/polygonal pore section. A possible hypothesis to explain this phenomenon is the confinement of the barrier layer in the small direction of the triangle, leading to an impossibility of etching the Al_2_O_3_ in this direction [38].

Finally, we show here that the quality of AAO template is widely improved compared to simple or double anodization processes, in terms of homogeneity of the array and pores, in term of size as well as in originality with arrays of oval pore section or double array of cylindrical/triangular pore shape [39]. The homogeneity in the parameters such as pore diameter or shape is useful to synthesise nanoobjects with a good regularity, whereas the no-defect triangular array brings a control over the localisation of these objects on a substrate. The high quality of the AAO obtained makes it very promising for nanofabrication.

### Silicon nanowires

Silicon nanowire (NW) arrays are widely studied nowadays because of their potential applications in microelectronics or detectors. Among the fabrication techniques, CVD is favoured. However, conventional techniques do not allow a good control on the position nor the homogeneity of the wires. Highly organised porous alumina has been successfully used as a template for the catalytic CVD growth of defect-free array of Si NW. For this, alumina is build on a <100> Si conductive wafer as described previously. Mould and anodization characteristics are adapted to the desired diameters, period and thickness of the future Si NW arrays.

**Figure 3:**
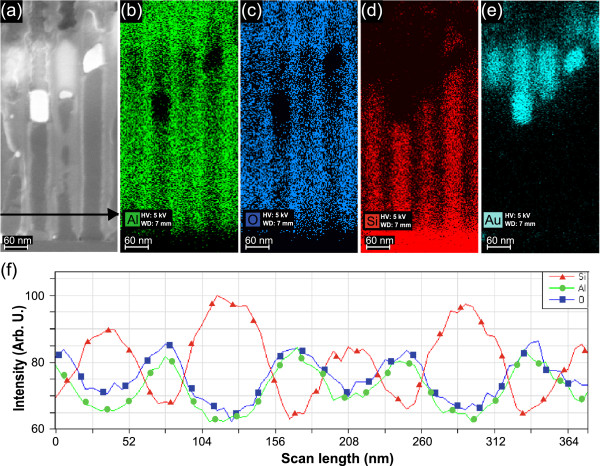
**Energy dispersive X-ray (EDX) analysis of Si NW. (a)** SEM image of the cross section, **(b)** aluminium cartography, **(c)** oxygen cartography, **(d)** silicon cartography, **(e)** gold cartography and **(f)** profile counts of oxygen, aluminium and silicon, along the arrow of **(a)**. The EDX analyses were conducted at 5-kV high voltage and for a 7-mm WD.

Energy dispersive X-ray analysis was performed on the cross section of the NW array before removal of the alumina template. High voltage of the electron beam of an ultra-Zeiss SEM was settled at 5 kV, and the sample was positioned at a working distance (WD) of 7 mm. Atoms of aluminium, oxygen, gold and silicon were mapped. Figure 3a,b,c,d,e shows the map of these atoms, and an intensity profile of Si, Al and O atoms is presented in Figure 3f. As expected, silicon is present in the template’s pores, the template is composed of aluminium and oxygen, and gold is present at the upper end of the silicon wires.

**Figure 4:**
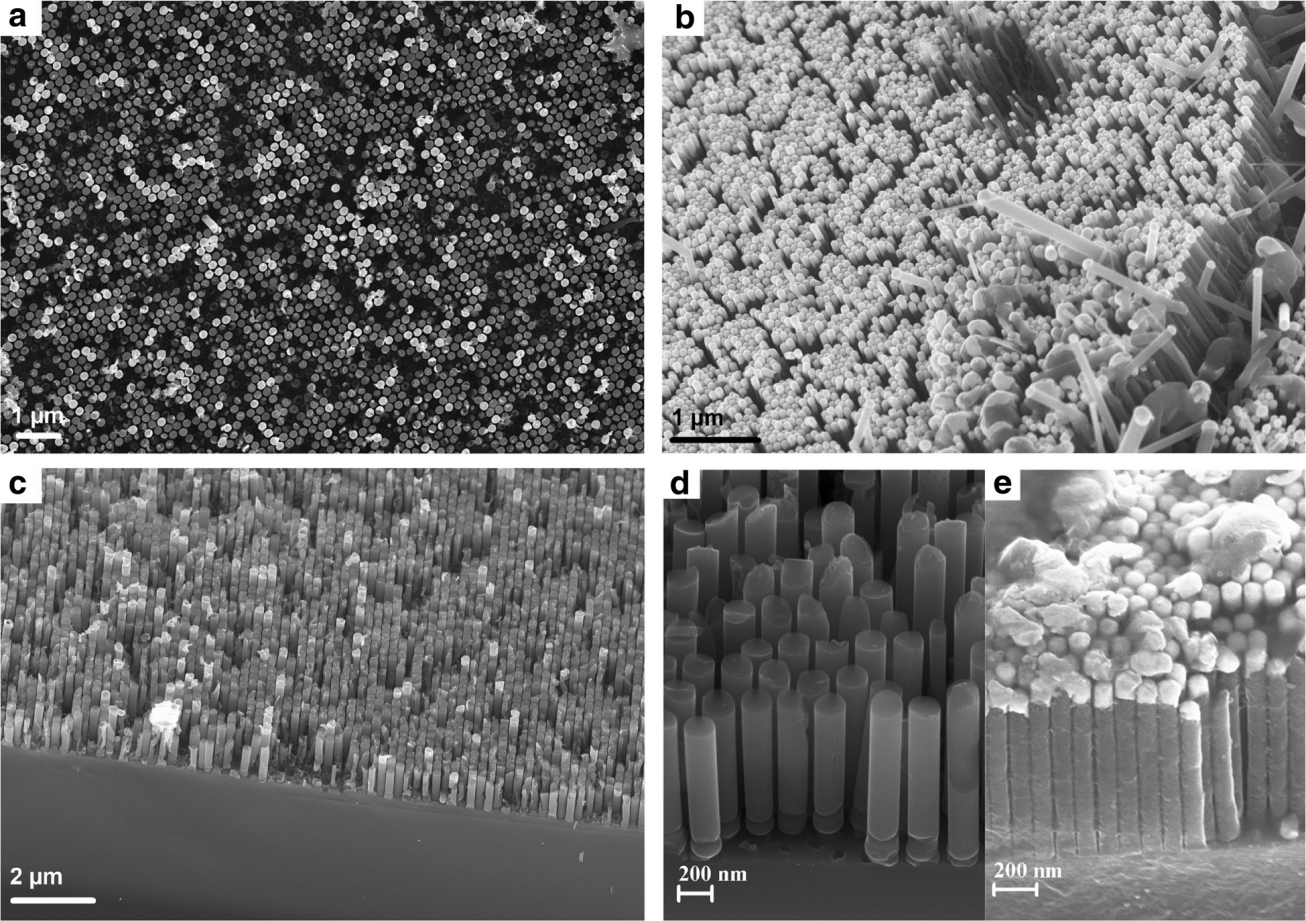
**Scanning electron micrographs of organised vertical silicon nanowire arrays grown on silicon substrates.** (**a**) Top view with the gold catalyst at the nanowires’ end. (**b**) Cross-sectional view with the gold catalyst at the nanowires’ end, with a nanowire diameter of 85 nm and period of 100 nm. (**c**) Cross-sectional view without the gold catalyst at the nanowires’ end, with a nanowire diameter of 190 nm and period of 250 nm. (**d**) Cross-sectional view without the gold catalyst at the nanowires’ end grown in alumina made with orthophosphoric acid, with a nanowire diameter of 190 nm and period of 250 nm. (**e**) Cross-sectional view of nanowires with gold catalyst grown in alumina made with oxalic acid, with a nanowire diameter of 85 nm and period of 100 nm.

Top view of silicon wires are reported in Figure 4a, showing a good filling rate around 80%. Different periods and diameters for the NWs are shown in Figure 4b,c,d,e, before or after the removal of the catalyst. One can notice the very good quality of the triangular lattice as well as the smooth cylindrical surface of the wires. On the foreground of Figure 4b, a few disordered wires have grown above the hexagonal array. Those wires are due to gold droplet coalescence above the alumina array. Indeed, when the wires reach the top surface of the alumina template, the gold droplets coalesce and nanowires with a bigger diameter grow above the array. As the <111> direction is the prefer orientation for NW growth [35] and because the growing conditions widely change outside the alumina, these nanowire kink with an angle of 54.7°. Besides, according to the homogeneity of the catalyst deposition, a difference in the speed of growth of the wires can be observed over the substrate between wires. It leads to small differences in the wires’ height, as shown in Figure 4d. In order to remove the parasite wires and obtain a perfectly homogeneous array, all wires are grown out of the alumina layer, and a method using ultrasound and plasma is used to selectively etch the wires above the alumina matrix [40].

Figure 4d,e shows a magnification of the flawless hexagonal array of Si in the case of growth in an alumina achieved in an orthophosphoric bath and an oxalic bath, respectively. One can notice that the wires at the interface are perfectly smooth and aligned in the case of oxalic acid, whereas we see the presence of a ring in the case of orthophosphoric acid. This is due to the intrinsic properties of the acid when the oxide layer reaches the silicon surface during anodization of alumina. This effect is less important than that of the oxalic acid. However, the walls of the nanowires are well defined and more regular with orthophosphoric acid than with oxalic acid, as can be seen in Figure 4d,e. One or the other acid should be chosen knowing these specific properties.

Due to the use of the AAO array, growth of silicon nanowires is possible even on non-preferential substrates. Indeed, the natural growth direction of the nanowires is the <111> direction using the VLS process. Here, thanks to the confinement in the pores, silicon nanowires are grown in the <100> direction, i.e. perpendicular to the surface of the most commonly used silicon wafer type in the microelectronics industry. Preliminary X-ray diffraction studies on the orientation of silicon nanowires obtained with a similar growth condition showed that both <100> and <111> orientations exist in the sample [40].

**Figure 5:**
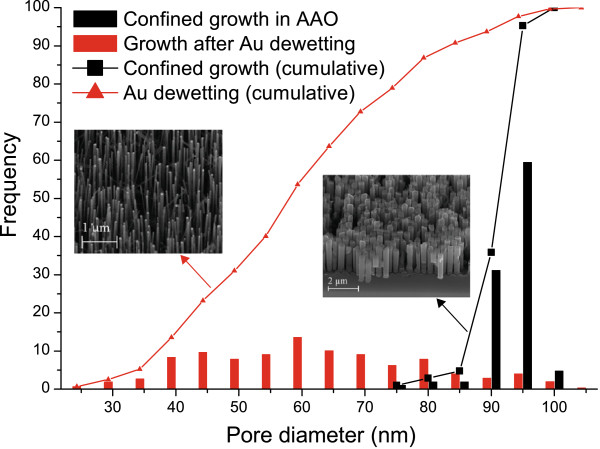
**Diagram of the diameter dispersions of the silicon nanowires, frequency and cumulative frequency.** Black: growth in AAO, red: growth using de-wetted gold.

Diagram of the distribution of the wires’ diameter in the case of a direct VLS growth with de-wetted catalyst drops [41] and in the case of a confined growth is presented in Figure 5. As expected, the size distribution decreases when using the AAO matrix to become even better than the one obtained for a growth from colloidal gold catalyst particles [42], i.e. standard deviation of Au de-wetted, 16.5 nm; confined growth in AAO, 3.9 nm; colloidal catalyst, 7.9 nm. Density is also improved with this method. Estimations show an increase by a factor of 60 in comparison with colloidal growth and by a factor of 1.16 compared to de-wetted growth, i.e. NW density for an average diameter of 100 nm: colloidal growth, 1.8 × 10^8^ cm^−2^[43]; de-wetting growth, 7.75 × 10^9^ cm^−2^; confined growth in AAO, 9 × 10^9^ cm^−2^.

To resume, the use of AAO as templates for the growth of Si nanowires drastically increases the quality of the final structures, specifically in terms of order on the substrate, density and diameter distribution.

## Conclusions

We report the successful preparation of hexagonal arrays of silicon nanowires on a <100> silicon substrate by CVD growth confined in flawless hexagonal porous alumina template. Large range of dimensions for the porous array is available: periods vary from 80 to 460 nm and diameters from 15 nm to any required diameter. Both oxalic and orthophosphoric acids give successful results. However, the walls of the pores are more regular with orthophosphoric acid, whereas the bottom of the pores presents fewer defects in the case of oxalic acid.

All process steps, demonstrated here on surfaces up to 2 × 2 cm^2^, are scalable to larger surfaces and compatible with microelectronic fabrication standards. Indeed, the catalyst, gold, can be replaced by copper, a metal more accepted by the semiconductor industry. The technique has been already developed in our team, for double anodization AAO, and will soon be implemented for nanoimprinted AAO [44]. The use of standard silicon wafers and the possibility to extend the presented process to wafer-scale areas at a reasonable cost (use of nanoimprint lithography) widen the number of possible applications.

Furthermore, in terms of integration, the confinement of nanowires in the AAO matrix is of great interest. Indeed, wires are electrically insulated from each other, and the high thermal and mechanical resistance of the alumina array can facilitate the implementation of further process steps.

Optimization of the formation of the guided pores - apparition of pores in between three imprinted ones - is a way to facilitate the mould fabrication and reduce its cost. Indeed, if the imprint of three pores leads to the creation of one more, a less dense array of pits is required for the mould, so with the same time of exposure, a larger surface of perfect porous alumina can be produced. If a densification of 1:4 in each direction would be possible, an increase of the area by a factor of 16 will be accessible, so 64 cm^2^ in our case, which is equivalent to 80% of the surface of a 4-in. wafer.

Further investigations are currently under progress to implement this type of nanowire arrays in photovoltaic devices, as recent results have shown a very high potential of organised silicon nanowire arrays for such applications [45].

## Abbreviations

AAO: Anodic aluminium oxide; CVD: Chemical vapour deposition; DSA: Directed self-assembly; FT: Fourier transforms; NIL: Nanoimprint lithography; NW: Nanowire; SEM: Scanning electron microscopy; VLS: Vapour-liquid–solid.

## Competing interest

The authors declare that they have no competing interest.

## Authors’ contributions

LD carried out the nanowires’ growth and the EDX analyses. PG participated in the CVD growth. MM carried out the nanoimprint mould fabrication and participated in its design. MZ participated in the nanoimprint process and the design of the nanoimprint mould. He participated in the redaction of the paper. DB participated in the porous anodic alumina fabrication and helped draft the manuscript. TG carried out the nanoimprint process, the anodization, the nanowire growth and the different analyses. She participated in the coordination of the study and in the redaction of the manuscript. All authors read and approved the final manuscript.
